# Recombinant SjP40 protein enhances p27 promoter expression in hepatic stellate cells via an E2F1-dependent mechanism

**DOI:** 10.18632/oncotarget.17248

**Published:** 2017-04-19

**Authors:** Yinong Duan, Lei Lyu, Dandan Zhu, Jianxin Wang, Jinling Chen, Liuting Chen, Chunzhao Yang, Xiaolei Sun

**Affiliations:** ^1^ Department of Pathogen Biology, School of Medicine, Nantong University, Nantong 226001, Jiangsu, People's Republic of China; ^2^ Laboratory Medicine Center, Affiliated Hospital of Nantong University, Nantong 226001, Jiangsu, People's Republic of China

**Keywords:** liver fibrosis, P40, p27, E2F1, Schistosoma japonicum

## Abstract

The p27 protein plays a critical role in cell cycle arrest. Our previous studies have demonstrated that recombinant P40 protein from *Schistosoma japonicum* (rSjP40) could induce G1 phase arrest of cell cycle. We, therefore, attempted to observe the effect of rSjP40 on p27 promoter activity in LX-2 cells and to explore its potential mechanisms in this study. Using both Western blot and dual-luciferase reporter assay, we demonstrated that rSjP40 could enhance the expression of p27 in LX-2 cells. Results obtained using truncated fragments of p27 promoter showed that rSjP40 increased p27 promoter activity in LX-2 cells, mainly via some transcription factors that bind to the -1740/-873 region of p27 promoter. Further studies confirmed that the enhancement of p27 promoter activity induced by rSjP40 was related to E2F1 in LX-2 cells. Transfection of siRNA of E2F1 could also restore the effect of rSjP40 on expression of p27 and partially on α-SMA. Therefore, our study provided further insights into the mechanism by which rSjP40 induces LX-2 cell cycle arrest at G1 phase and inhibits HSC activation. Our results provide basis for future study of the blocking effect of rSjP40 in liver fibrosis.

## INTRODUCTION

Liver fibrosis is a common pathological process observed in various chronic liver diseases [1]. During liver fibrosis, the dominant characteristic of hepatic stellate cells (HSCs) activation triggers the production of abundant extracellular matrix (ECM) proteins, including α-smooth muscle actin (α-SMA) and collagen proteins [2]. Schistosome is one of the pathogenic factors that induce liver fibrosis. Sachistosome infection often leads to schistosomiasis in patients [3]. Interestingly, previous studies have shown that eggs and soluble egg antigens (SEA) from *Schistosoma japonicum* (*S. japonicum*) and *Schistosoma mansoni* (*S. mansoni*) could restrain the proliferation and activation of HSCs [4–6]. We have previously defined that recombinant P40 protein from *S. japonicum* (rSjP40), a dominant component of SEA, could also suppress activation of HSCs and accelerate senescence of HSCs through the STAT3/p53/p21 pathway [7].

The p27 protein is a structural homologue of the p21 protein. It has been demonstrated to play a critical role in cell cycle arrest [8]. More specifically, it has been reported that p27 is the key regulator of G0/G1 phase arrest and cell proliferation suppression in platelet-derived growth factor (PDGF)-BB-activated HSCs through repression of the PI3K/Akt/ FOXO3a pathway [9]. Since our previous studies demonstrated that SEA and its component P40 protein from *S. japonicum* could induce G1 phase arrest of cell cycle [4, 7, 10], we aimed to further examine the effect of rSjP40 on the activity of p27 promoter in LX-2 cells and to explore its potential mechanisms in this study.

## RESULTS

### Expression of p27 was enhanced in rSjP40-stimulated LX-2 cells

**Figure 1:**
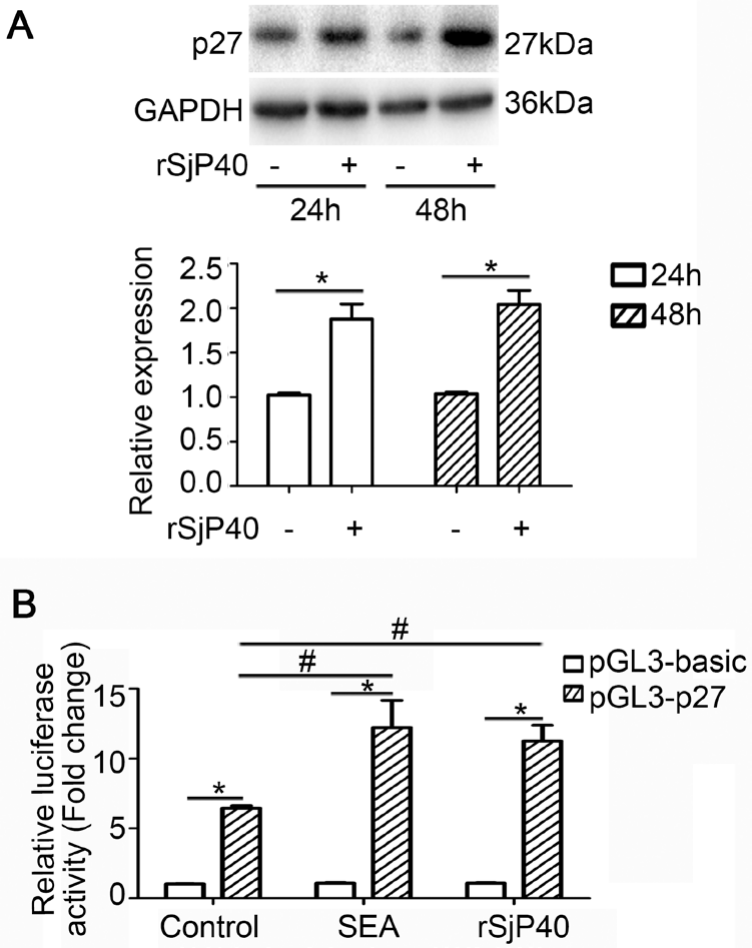
p27 expression was up-regulated in LX-2 cells treated with rSjP40 (**A**) p27 protein expression levels in LX-2 cells treated with rSjP40 at the concentration of 20 μg/mL were evaluated by Western blot. *P < 0.05, compared to each untreated group. (**B**) p27 promoter fluorescence activities were elevated in LX-2 cells treated with rSjP40 or SEA. *P < 0.05, compared to each pGL3-basic group. ^#^*P* < 0.05, compared to untreated group transfected with pGL3-p27. The data are presented as the mean ± SEM of at least three independent experiments.

It has been demonstrated that p27 can suppress cell cycle progress through arresting cells in the G0/G1 phase by inhibiting the expression and activity of cyclin D1 [11, 12]. Previously, we have revealed that LX-2 cells were arrested at G1 phase after rSjP40-stimulation [7]. In this study, we further observed that rSjP40 could increase the protein expression level of p27 in LX-2 cells treated with rSjP40 for 24 h and 48 h (Figure 1A). Results from luciferase activity analysis also confirmed that p27 promoter activities were distinctly induced in LX-2 cells stimulated with rSjP40 or SEA for 24 h (Figure 1B). These results suggested that rSjP40 could promote p27 expression in LX-2 cells.

### rSjP40 increased p27 promoter activity in LX-2 cells, mainly via transcription factors that bind to -1740/-873 region of the p27 promoter

**Figure 2:**
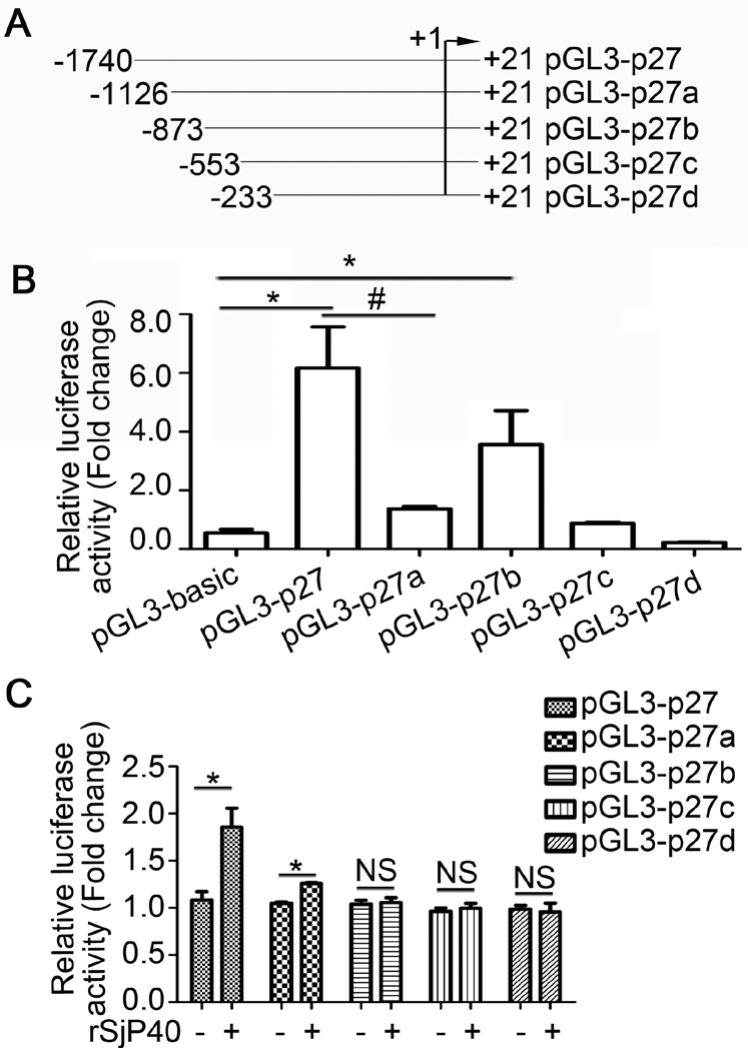
rSjP40 increased p27 promoter activity in LX-2 cells via transcription factors that bind to the -1740/-873 region of the p27 promoter (**A**) Diagram of the construction of p27 promoter truncated fragments. (**B**) Fluorescence activities of pGL3-basic, pGL3-p27, pGL3-p27a, pGL3-p27b, pGL3-p27c and pGL3-p27d in LX-2 cells were determined by dual-luciferase reporter assay. *P < 0.05, compared to pGL3-basic group. ^#^*P* < 0.05, compared to pGL3-p27 group. (**C**) LX-2 cells were treated with or without rSjP40. Fluorescence activities of pGL3-p27, pGL3-p27a, pGL3-p27b, pGL3-p27c and pGL3-p27d were determined by dual-luciferase reporter assay. *P < 0.05, compared to each untreated group. NS, *P* > 0.05, no significant difference was found. The data are presented as the mean ± SEM of at least three independent experiments.

In order to narrow down the activity region, we established four luciferase reporter plasmids of truncated fragments of the p27 promoter, pGL3-p27a, pGL3-p27b, pGL3-p27c, pGL3-p27d (Figure 2A). As shown in Figure 2B, transcription factors could promote p27 promoter activity by binding to the -1740/-1126 region of the p27 promoter. Meanwhile, transcription factors that could inhibit p27 promoter activity may bind to the -1126/-873 region of the p27 promoter (Figure 2B).

To explore the mechanism through which rSjP40 could promote p27 promoter activity, the truncated fragments were then transfected into rSjP40-stimulated LX-2 cells, respectively. Results from dual-luciferase reporter assay showed that p27 promoter activity was apparently increased in rSjP40-treated LX-2 cells transfected with pGL3-p27 and pGL3-p27a (Figure 2C) and that rSjP40 might have increased p27 promoter activity in LX-2 cells via some transcription factors that bound to the -1740/-873 region of the p27 promoter.

### Transcription factor E2F1 targeted the -1740/-873 activity region of p27 promoter

**Figure 3:**
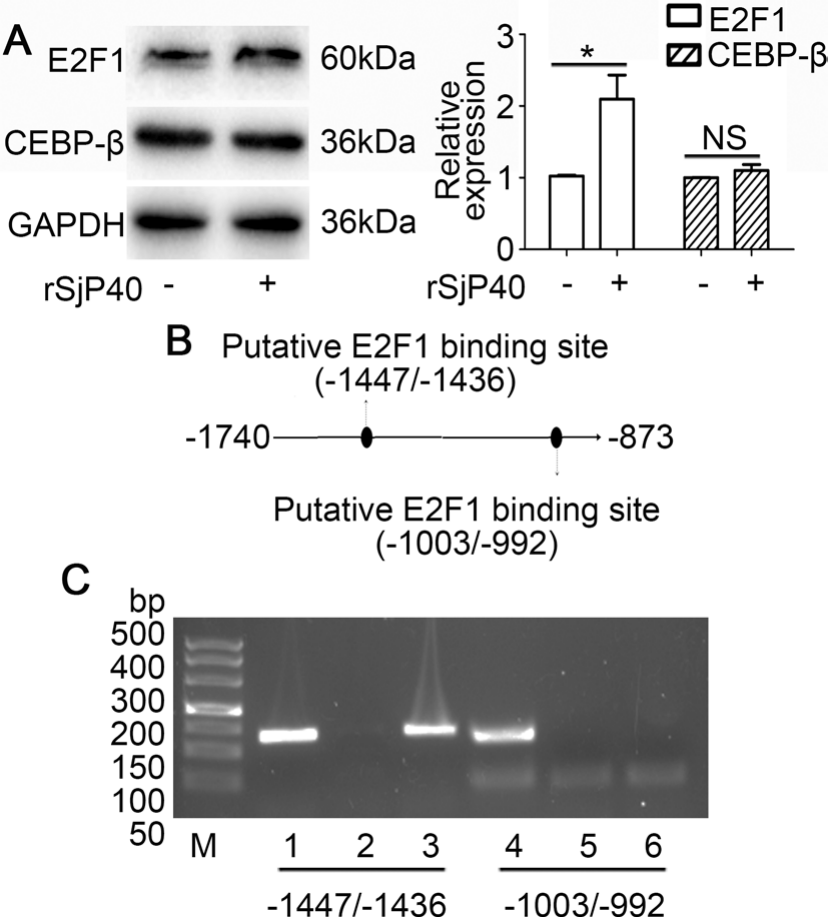
Transcription factor E2F1 binds to the -1740/-873 activity region of the p27 promoter (**A**) Protein expression of E2F1 and CEBP-β after rSjP40 treatment as investigated by Western blot. *P < 0.05, compared to untreated group. NS, *P* > 0.05, no significant difference was found. (**B**) Diagram of E2F1 binding sites in the -1740/-873 activity region of the p27 promoter. (**C**) CHIP analysis was performed to confirm the binding of E2F1 to the p27 promoter. Lanes 1 and 4: input group. Lanes 2 and 5: normal IgG group. Lanes 3 and 6: anti-E2F1 group.

Transcription factor binding sites in the -1740/-873 activity region were predicted using p-match and JASPAR database. Both software tools were employed using the default settings. The prediction results of JASPAR database showed that two E2F1 binding sites (-1447/-1436, -1003/-992), four CEBP-β binding sites and seven STAT3 binding sites may be present in the activity region. However, results from p-match database indicated that only one E2F1 (-1447/-1436) and one NKX2-5 binding sites are located within the -1740/-873 region of p27 promoter. We, therefore, speculated that rSjP40 may promote p27 promoter activity in LX-2 cells through an E2F1-dependent mechanism. Furthermore, we observed that rSjP40 could induce E2F1 expression, but not CEBP-β expression, in LX-2 cells by Western blot (Figure 3A). To investigate whether the two binding sites are effective, two pairs of primers were designed for CHIP. Results from CHIP experiment confirmed that E2F1 could bind to the p27 promoter at the -1447/-1436 location (Figure 3B and Figure 3C). These results preliminarily indicated that the effect of rSjP40 on p27 promoter activity in LX-2 cells may be attributed to E2F1.

### rSjP40-mediated enhancement of p27 promoter activity was related to E2F1 in LX-2 cells

**Figure 4:**
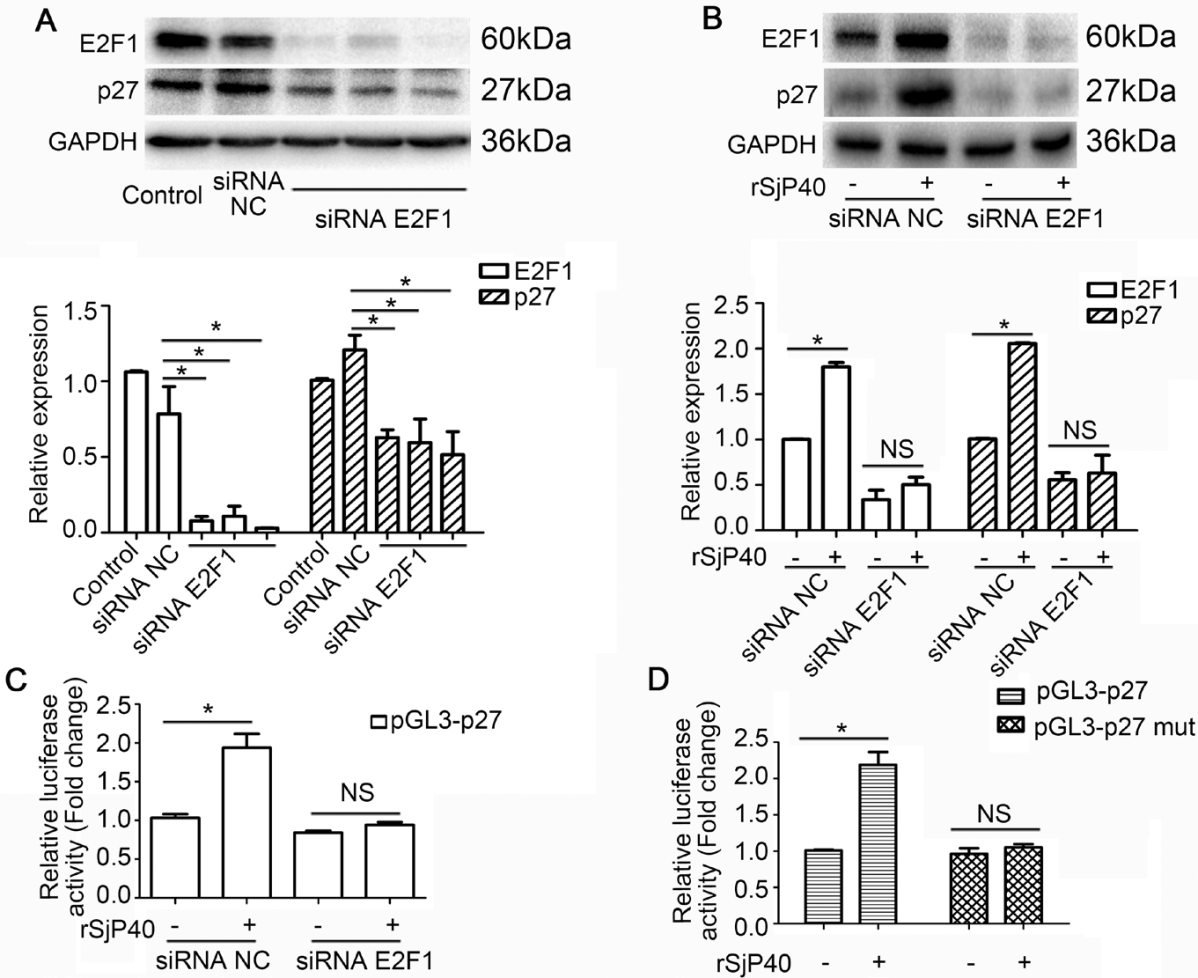
rSjP40 mediated enhancement of p27 promoter activity was related to E2F1 in LX-2 cells (**A**) Relationship between E2F1 and p27 protein expression. *P < 0.05, compared to siRNA NC- group. (**B** and **C**) Western blot analysis and dual-luciferase reporter assay were conducted respectively to examine the effect of E2F1 on p27 expression in LX-2 cells treated with rSjP40. *P < 0.05, compared to rSjP40-siRNA NC+ group. NS represents *P* > 0.05, between rSjP40-siRNA E2F1 + group and rSjP40 + siRNA E2F1 + group. (**D**) Dual-luciferase reporter assay was performed to observe the effect of rSjP40 on the activity of pGL3-p27 and pGL3-p27 mut in LX-2 cells. *P < 0.05, compared to rSjP40-pGL3-p27+ group. NS represents *P* > 0.05, between rSjP40-pGL3-p27 mut + group and rSjP40 + pGL3-p27 mut + group. The data are presented as the mean ± SEM of at least three independent experiments.

Next, we explored whether the specific knockdown of endogenous E2F1 could impact p27 expression in LX-2 cells. As shown in Figure 4A, transfection with siRNA E2F1 led to reduction of p27 protein expression. Transfection with siRNA E2F1 also eliminated rSjP40-induced promotion of p27 protein expression (Figure 4B) and p27 promoter activity (Figure 4C). However, rSjP40 could not induce the activity of a mutant p27 promoter, which contained a mutation in the E2F1 binding site (Figure 4D). All the above results suggested that rSjP40 may promote p27 expression level in LX-2 cells via an E2F1-dependent mechanism.

### rSjP40-mediated inhibition of α-SMA expression was partially related to E2F1 in LX-2 cells

**Figure 5:**
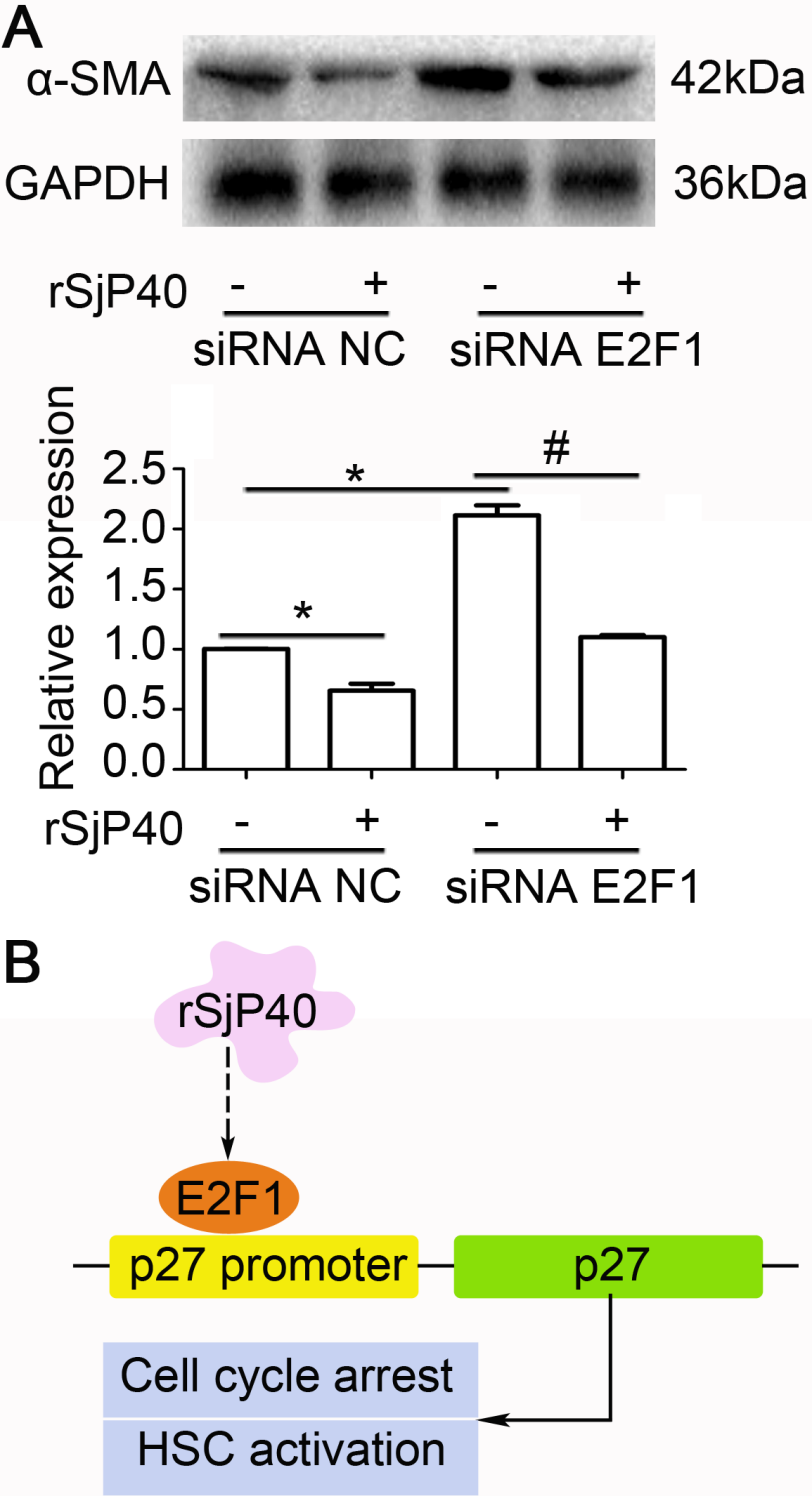
Inhibition of α-SMA expression mediated by rSjP40 was related to E2F1 in LX-2 cells (**A**) Effect of E2F1 on α-SMA expression in LX-2 cells treated with rSjP40 was detected by Western blot. *P < 0.05, compared to rSjP40-siRNA NC+ group. ^#^*P* < 0.05, compared to rSjP40-siRNA E2F1+ group. (**B**) Potential mechanism of rSjP40-mediated inhibition on activation of LX-2 cells was shown. rSjP40 protein may inhibit activation of LX-2 cells via a p27 and E2F1 dependent mechanism.

Since rSjP40 could inhibit α-SMA expression in LX-2 cells [7, 13], we asked whether siRNA of endogenous E2F1 could impact α-SMA expression in LX-2 cells. As shown in Figure 5A, α-SMA expression could be up-regulated in rSjP40-siRNA E2F1+ group, compared to that in rSjP40-siRNA NC+ group (*P < 0.05). However, which is different from p27 and E2F1 expression, significant difference could be found between the rSjP40-siRNA E2F1+ group and rSjP40+siRNA E2F1+ group (^#^*P* < 0.05). These results showed that transfection with siRNA E2F1 could partially reverse the inhibitory effect of rSjP40 on α-SMA expression. Therefore, we concluded that rSjP40 may inhibit HSC activation through an E2F1/p27 pathway (Figure 5B).

## DISCUSSION

Inflammatory granuloma formation and liver fibrosis are characteristics of schistosomiasis [14]. During liver fibrosis in schistosomiasis, HSC activation is one of main events of liver fibrosis, and is responsible for collagen production and α-SMA accumulation. This developmental process of HSCs often leads to fibrogenesis in the liver of patients with schistosomiasis [15]. Hence, the inhibition of HSC activation and the induction of HSC apoptosis and senescence are often employed as the main strategies for treating liver fibrosis [16]. As previously described, cell cycle arrest at G0/G1 phase of HSCs may be an important mechanism through which potential therapeutics could block liver fibrosis. For example, An et al. have demonstrated PTEN could induce arrest at the G0/G1 and G2/M phase in both human LX-2 cells and rat primary HSCs [17]. Bohanon et al. also found that CYD0692, which is a newly designed analog of Oridonin, could induce LX-2 cell cycle arrest at S phase [18].

Although it is traditionally believed that schistosome eggs are the cause of liver fibrosis in schistosomiasis, Anthony et al. first demonstrated that eggs from *S. mansoni* could inhibit the activation of LX-2 cells directly, accompamied by an increased accumulation of lipid droplets in LX-2 cells [5]. They also reported that eggs from *S. japonicum* were able to exert an anti-fibrotic effect on LX-2 cells [6]. Based on their novel findings, we further demonstrated that SEA from *S. japonicum* may block liver fibrosis by inducing HSC apoptosis and senescence [4, 10]. In addition, the anti-activating effect of SEA was confirmed both in HSCs activated *in vivo* and *in vitro* [4]. P40 protein from *S. japonicum* (SjP40) and *S. mansoni* (SmP40) is the main component of SEA. SmP40 has been described as a mediator associated with reducing collagen deposition and fibrosis [19]. Recently, we also identified that rSjP40 could inhibit TGF-β1-induced activation of LX-2 cells through the TGF-β1/Smads pathway [13]. Interestingly, rSjP40 is different from SEA and could not induce cell apoptosis in LX-2 cells [20]. Importantly, we found that the anti-fibrotic effects of SEA and rSjP40 both manifested simultaneously with the induction of cell cycle arrest. As reported, SEA may induce cell cycle arrest at the G1 phase and inhibit HSC proliferation *in vitro* [4, 10]. rSjP40 also can also induce arrest at the G1 phase and induce cell senescence in LX-2 cells [7]. Therefore, we speculated that SEA and rSjP40 may block liver fibrosis by arresting HSCs at the G1 phase, inhibiting HSC activation and inducing HSC senescence.

Previous studies showed that p27 protein could participate in the arrest process of the cell cycle and cell proliferation [8]. As Egozi et al. reported, p27 could negatively regulate the activity of protein kinase complex cyclin E-CDK2, thereby blocking cell cycle progression from G1 to S phase [21]. Eukaryotic translation initiation factor 3a (eIF3a)/p27 pathway is considerably essential for TGF-β1-induced cell proliferation in rats pulmonary fibroblasts. L-mimosine treatment could up-regulate p27 expression and attenuate pulmonary fibrosis [22]. In liver fibrosis, fluorofenidone, a pyridine agent, could alleviate liver fibrosis by inhibiting HSC activation and proliferation. The potential mechanism of this blocking effect of fluorofenidone may be attributed to its enhancive effect on p27 expression, which was associated with G0/G1 phase cell cycle arrest of fluorofenidone-treated HSCs [23]. In our previous studies, we also confirmed that SEA and rSjP40 could induce HSC senescence, which manifested as cell cycle arrest of HSCs, partly through the up-regulation of p27 expression [20, 24]. In this study, we further demonstrated that both SEA and rSjP40 could enhance the activity of p27 promoter.

It has been established that E2F1 is also involved in the regulation of cell cycle, and the pRb2/HDAC1/E2F1 complex may negatively regulate cell cycle regulatory proteins, including Cdk2/4 and cyclin D3/E [25]. The transcription factor E2F1 can bind to the p27 promoter and subsequently, enhance p27 expression [26]. In addition, a negative feedback mechanism was observed between p27 and E2F1, as inhibition of endogenous p27 expression could further promote E2F1 functions in H1299 cells through pRb [26]. In our study, we found that rSjP40 could induce p27 and E2F1 expression simultaneously. Further study demonstrated that the reduction of E2F1 by siRNA could block the rSj40-induced upregulation of p27 expression. Meanwhile, mutations in the E2F1 binding site of the p27 promoter could also block the rSjP40-induced up-regulation of p27 promoter activity. However, siRNA of E2F1 could partially restore the effect of rSjP40 on the inhibition of α-SMA expression and we speculated some other signals may exist in rSjP40-inhibited HSC activation.

In conclusion, we concluded that rSjP40 protein may enhance p27 promoter activity in LX-2 cells via an E2F1-dependent mechanism, which may further induce HSC cell cycle arrest and partially inhibit the activation of HSCs (Figure 5B).

## MATERIALS AND METHODS

### Cell culture and treatment

LX-2 cells, a human HSC line, preserved in our lab [27], were cultured in Dulbecco's Modified Eagle's Medium (DMEM, Gibco, USA) supplemented with 10% of fetal bovine serum (FBS), in a humidified atmosphere containing 5% CO_2_ at 37°C. LX-2 cells were inoculated into 6 well culture-plates and treated with rSjP40 at the concentration of 20 μg/mL. rSjP40 was obtained as previously described [13].

### Bioinformatics analysis of p27 promoter and construction of plasmids containing p27 promoter sequence

The sequence of the p27 promoter (−1737 bp to +24 bp), encompassing the ATG start site, was obtained from the National Center for Biotechnology Information (NCBI, http://www.ncbi.nlm.nih.gov/). Transcription factor binding sites were predicted using the JASPAR network tool software (http://jaspar.genereg.net/) and p-match network platform (http://www.gene-regulation.com/pub/programs.html#pmatch). To construct p27 promoter associated plasmids, the polymerase chain reaction (PCR) primers were designed (Table 1). Genomic DNA was extracted from LX-2 cells according to instructions for the QIAamp^®^ DNA Micro Kit (Qiagen, Germany). Promoter fragments of p27 from −1737 bp to +24 bp was amplified from genomic DNA template, cloned into pGL3-basic vector (Promega, USA) and renamed as pGL3-p27. Then, four luciferase reporter plasmids containing truncated fragment of p27 promoter and one plasmid containing the mutant E2F1-binding site were generated separately using corresponding PCR primers shown in Table 1, and were named as pGL3-p27a, pGL3-p27b, pGL3-p27c, pGL3-p27d and pGL3-p27 mut, respectively.

**Table 1:** Primers used in this study

primer	Sequence (5′→ 3′)	Purpose
p27 F^1^	GCCCTGCTCATCGTCCTACTTTAC	pGL3-p27
p27a F	CGTTCGCTTTGGCTTCTTCCCT	pGL3-p27a
p27b F	CGGTCCTCTGGTCCAGGTCC	pGL3-p27b
p27c F	CGCCGCAACCAATGGATCTC	pGL3-p27c
p27d F	TCGCCAGTCCATTTGATCAGC	pGL3-p27d
p27 R^2^	GTTAGACACTCGCACGTTTGACATC	Reporters^3^
p27-mut F1	GCCCTGCTCATCGTCCTACTTTACCTTC	pGL3-p27 mut
p27-mut R1	GAGTGTGCGATGTAGATACAACAGCTCCTTCC	pGL3-p27 mut
p27-mut F2	TGTATCTACATCGCACACTCAGGTAGAGGAAA	pGL3-p27 mut
p27-mut R2	GTTAGACACTCGCACGTTTGACATC	pGL3-p27 mut
E2F1(a) F	GTTGGAGCAGTGAAATCTGGTGAG	ChIP
E2F1(a) R	AACTCGTCCCTTTCTACTTTTCTG	ChIP
E2F1(b) F	GAACCATTGCCCACTGCCTC	ChIP
E2F1(b) R	ACCTCGTGGTCTGCGGGGGA	ChIP

^1^F: Forward.

^2^R: Reverse.

^3^Reporters: pGL3-p27, pGL3-p27a, pGL3-p27b, pGL3-p27c, pGL3-p27d.

### Dual-luciferase reporter assay

Reporter plasmids were transiently transfected into LX-2 cells according to the manufacturer's instructions for FuGENE Transfection Reagent (Promega, USA). After transfection for 12 h, rSjP40 were added and LX-2 cells were cultured for another 24 h. Then the cells were harvested for luciferase activity analysis on a luminometer following the instructions of the Dual-luciferase Reporter Assay system (Promega, USA).

### E2F1 interference experiment

LX-2 cells were seeded in a 6-well plate at 2 × 10^5^ cells/well in 2 mL of medium without antibiotic/antimycotic and cultured to 70–90% confluency before transfection. Then 5 μL of lipofectamine 2000 reagent (Invitrogen, USA) was diluted with 500 μL of DMEM and incubated for 5min at room temperature. Then, 5 μL of siRNA E2F1 (Sigma, USA) or negative control siRNA (siRNA NC, Sigma, USA) were added to the lipofectamine 2000/medium mixture and incubated for 20 min at room temperature. Next, the mixture was added to 2 mL of cell culture media in the 6-well plate. After transfection for 6 h, the culture medium was discarded and replaced with medium containing rSjP40 at a concentration of 20 μg/mL for another 24 h.

### Chromatin immunoprecipitation (ChIP)

ChIP experiments were performed using SimpleChip Kit (Cell Signaling Technology, USA). Anti-E2F1 antibody was purchased from Santa Cruz Biotechnology (USA) and was used to precipitate the DNA-protein complex. Normal IgG provided in SimpleChip Kit was used as the negative control. Purified DNA obtained from SimpleChip Kit was then used as the template and PCR was conducted using two pairs of primers (Table 1), which were designed based on the different E2F1-binding sites of the p27 promoter.

### Western blot

Whole protein samples were obtained from lysates of LX-2 cells in RIPA buffer (Beyotime, China) and subjected to 10% sodium dodecyl sulfate-polyacrylamide gel electrophoresis (SDS-PAGE). The proteins were then transferred to 0.45 μm polyvinylidene fluoride (PVDF) membranes (Merck, Germany). The membranes were then incubated at 4°C for 12 h with primary antibodies including p27 (Santa Cruz, USA), E2F1 (Santa Cruz, USA), α-SMA (Santa Cruz, USA) and GAPDH (Goodhere, China). Then, the membranes were washed and incubated with horseradish peroxidase (HRP)- conjugated secondary antibodies (Santa Cruz Biotechnology, USA) and visualized with ECL-chemiluminescent kit (Merck, Germany).

### Statistical analysis

The data are presented as the mean ± SEM of at least three independent experiments. Data were analyzed using one-way ANOVA method or Independent Samples *T*-test in SPSS 15.0. Differences were considered significant if *P* < 0.05.
